# Impact of evidence-based nursing interventions on postoperative recovery and complications after endovascular aneurysm intervention: A retrospective cohort study

**DOI:** 10.1097/MD.0000000000049646

**Published:** 2026-07-03

**Authors:** Zhifei Zhang, Jianjun Gu, Lili Su, Yan Chen, Mengmeng Li, Mengke Liu

**Affiliations:** a Department of International Medical Center, Henan Provincial Key Medicine Laboratory of Nursing, Henan Provincial People’s Hospital; Zhengzhou University People’s Hospital, Zhengzhou, Henan, China.

**Keywords:** complications, endovascular aneurysm intervention, evidence-based nursing, intensive care unit, postoperative recovery

## Abstract

Endovascular aneurysm intervention is increasingly used for aortic and peripheral aneurysmal disease, yet postoperative recovery may be delayed by access-site events, cardiopulmonary instability, symptom burden, and renal vulnerability related to iodinated contrast exposure. This single-center retrospective cohort study evaluated the association between an evidence-based nursing bundle and postoperative outcomes in adults undergoing endovascular aneurysm intervention between January 2024 and December 2025. Patients treated in 2024 received routine perioperative nursing (control, n = 68), whereas those treated in 2025 received a standardized evidence-based nursing program (intervention, n = 69). Baseline demographic, clinical, procedural, and preoperative laboratory characteristics were comparable between groups. The intervention group demonstrated shorter postoperative length of stay (6.04 ± 1.62 vs 7.52 ± 1.91 days; *P* <.001) and lower intensive care unit admission (11.6% vs 26.5%; *P* = .026), with reduced ICU duration among admitted patients (median 1.08 vs 2.59 days; *P* = .013). Recovery milestones occurred earlier in the intervention group, including first ambulation, oral intake, flatus, and defecation (all *P* ≤ .023). Symptom control improved, with lower Numeric Rating Scale pain scores at 6, 24, and 48 hours (all *P* <.001) and reduced postoperative nausea and vomiting (14.5% vs 32.4%; *P* = .013). Any postoperative complication occurred less frequently with evidence-based nursing (17.4% vs 35.3%; *P* = .017). Postoperative serum creatinine change was smaller (*P* = .021), while contrast-associated acute kidney injury was numerically lower (*P* = .097). Evidence-based nursing was associated with accelerated recovery, improved symptom control, and reduced overall complications after endovascular aneurysm intervention.

## 1. Introduction

Endovascular aneurysm intervention has become a central therapeutic approach for a wide spectrum of aortic and peripheral aneurysmal diseases, driven by its minimally invasive profile and expanding device capabilities for anatomically complex pathology. Contemporary endovascular practice increasingly includes advanced or complex repairs, and large clinical series continue to document meaningful perioperative morbidity despite high technical success, particularly among older patients with substantial cardiometabolic and renal comorbidity burdens.^[1,2]^ In parallel, perioperative management is evolving, with recent vascular-focused reviews emphasizing that outcomes after aneurysm repair are influenced not only by procedural technique but also by structured perioperative care domains such as monitoring strategies, fluid and transfusion practices, infection prevention, and standardized recovery pathways.^[3]^ Although enhanced recovery after surgery (ERAS) frameworks have demonstrated benefit across multiple surgical specialties, the vascular surgery evidence base remains comparatively heterogeneous. A recent evidence map and scoping review highlighted both the breadth of ERAS components tested in vascular populations and persistent gaps regarding consistent implementation and patient-centered outcomes.^[4]^ Qualitative research in complex endovascular aortic repair further indicates that recovery is shaped by communication quality, symptom management, and coordinated mobilization and discharge processes – domains in which nursing practice has a central operational role.^[5]^ Early ambulation, a core ERAS element, has also been linked to shorter functional recovery trajectories after major aortic surgery, supporting the clinical rationale for nursing-led mobilization targets and milestone-driven rehabilitation.^[6]^

Postoperative recovery after endovascular aneurysm intervention is frequently complicated by issues that are amenable to standardized nursing processes, including access-site events, cardiopulmonary instability, nausea and vomiting, delayed gastrointestinal recovery, and pain that can impede mobilization. Updated consensus guidance on postoperative nausea and vomiting underscores multimodal, risk-stratified prevention and structured reassessment, reinforcing the importance of protocolized perioperative symptom control.^[7]^ In addition, iodinated contrast exposure and baseline renal vulnerability make kidney protection a salient safety priority. Recent cardiovascular and radiology statements have refined the conceptual framework of contrast-associated acute kidney injury (AKI) and emphasize risk stratification, judicious contrast dosing, and standardized prophylactic measures.^[8]^ Given that chronic kidney disease is prevalent in aneurysm populations and is strongly prognostic for adverse outcomes, contemporary international guidance on chronic kidney disease evaluation and management further supports systematic assessment of baseline renal function to inform perioperative planning.^[9]^

Collectively, these considerations provide a compelling basis for evaluating evidence-based, nursing-delivered perioperative protocols tailored to endovascular aneurysm intervention, with an emphasis on accelerating recovery milestones, improving symptom control, and reducing preventable postoperative complications in real-world practice.

## 2. Methods

### 2.1. Study design

This study was approved by the Ethics Committee of Henan People’s Hospital. This single-center retrospective cohort study consecutively enrolled patients who underwent endovascular aneurysm intervention at our institution between January 2024 and December 2025 to evaluate the impact of evidence-based nursing interventions on postoperative recovery and complication profiles. Patients treated from January to December 2024 received routine perioperative nursing care and were assigned to the control group, whereas those treated from January to December 2025 received a standardized evidence-based nursing program and were assigned to the intervention group. Eligible participants were adults (≥18 years) with imaging-confirmed aneurysmal disease who completed the index endovascular procedure (e.g., endovascular aneurysm repair) and had complete perioperative nursing records and outcome documentation sufficient for assessment of postoperative recovery parameters and predefined complications. Exclusion criteria comprised: non-aneurysmal pathology or concomitant major procedures not attributable to the endovascular aneurysm intervention (e.g., hybrid or open conversion during the index admission); preexisting severe infection, septic shock, or other critical systemic conditions that could substantially confound postoperative inflammatory or recovery trajectories; end-stage organ failure with expected short-term mortality independent of the procedure (e.g., end-stage renal disease on maintenance dialysis, decompensated cirrhosis, or advanced malignancy with limited life expectancy); and inability to evaluate primary outcomes due to early transfer to another facility, discharge against medical advice, or missing key perioperative data. The study was conducted in accordance with the Declaration of Helsinki and was approved by the institutional medical ethics committee. Written informed consent was obtained from all participants in accordance with institutional requirements.

### 2.2. Perioperative nursing protocols for endovascular aneurysm intervention

Routine perioperative nursing (control group) followed the department’s standard pathway for patients undergoing endovascular aneurysm intervention. Before the procedure, nurses completed admission assessment and routine preoperative preparation, including verification of identity and consent, medication reconciliation, fasting and skin preparation, establishment of venous access, and baseline evaluation of vital signs, pain, and limb perfusion. General perioperative education was provided regarding the procedural process and basic postoperative precautions. After the intervention, patients received scheduled monitoring of hemodynamics and oxygenation, pain assessment with administration of prescribed analgesics and antiemetics, and routine surveillance of the puncture site for bleeding, hematoma, and dressing integrity. Peripheral neurovascular status of the access-side limb was assessed at regular intervals. Intake and output were recorded, early mobilization was encouraged as tolerated, and routine discharge education addressed wound care, medication adherence, activity restriction, follow-up arrangements, and indications for seeking urgent medical attention.

Evidence-based nursing (intervention group) was delivered as a standardized, protocol-driven bundle to optimize recovery and reduce procedure-related complications. Preoperatively, nurses performed structured risk screening for bleeding, thromboembolism, renal impairment, infection, delirium, and uncontrolled blood pressure, and communicated identified risks to the treating team to support individualized perioperative planning. Patient education was standardized using a brief comprehension check to ensure understanding of access-site precautions, early warning symptoms, and postoperative activity goals. Postoperatively, monitoring was intensified during the early phase and aligned with predefined escalation criteria for abnormalities in blood pressure, oxygen saturation, urine output, mental status, puncture-site findings, or distal perfusion. Targeted preventive measures included reinforced access-site protection and graded activity guidance to mitigate bleeding and hematoma; standardized neurovascular surveillance for early recognition of limb ischemia; hydration and strict fluid-balance management to reduce contrast-associated kidney injury risk; early pulmonary care and progressive mobilization to reduce atelectasis and pneumonia; and implementation of mechanical and prescribed pharmacologic thromboprophylaxis with adherence checks. Delirium prevention focused on orientation, sleep hygiene, and timely pain control. Discharge readiness was evaluated against standardized criteria, and structured education emphasized medication safety (including antiplatelet or anticoagulant regimens), access-site care, activity restrictions, symptom-triggered return instructions, and early post-discharge contact when feasible.

### 2.3. Data collection

Data were retrospectively extracted from the institutional electronic medical record, perioperative nursing documentation system, anesthesia records, operative reports, and laboratory information system for all eligible patients who underwent endovascular aneurysm intervention between January 2024 and December 2025. Using a standardized data abstraction form developed before data collection. Two trained reviewers, blinded to the study hypothesis and group allocation during the abstraction process, independently reviewed electronic records. Variables were cross-checked against source documents. Outcomes were defined a priori using standardized clinical criteria: postoperative nausea and vomiting was defined as nursing documentation of emesis or rescue antiemetic use; pain was quantified using the validated Numeric Rating Scale at scheduled nursing assessments; and complications were classified according to Clavien–Dindo grading and KDIGO definitions for AKI.

Baseline demographic and lifestyle variables included age, sex, body mass index, current smoking status, and current alcohol use at admission. Clinical characteristics comprised preexisting comorbidities documented in the medical history or discharge diagnoses, including hypertension, diabetes mellitus, coronary artery disease, chronic kidney disease, and chronic obstructive pulmonary disease or other chronic lung disease. Aneurysm- and procedure-related variables were obtained from imaging reports and operative records, including aneurysm type/location, maximum aneurysm diameter, access approach (percutaneous vs surgical cutdown), anesthesia type (general vs regional/local), operative time, contrast volume, and estimated blood loss. Preoperative laboratory and physiologic indices were collected from the closest measurements within 24 hours before the procedure, including hemoglobin, white blood cell count, serum creatinine, estimated glomerular filtration rate, and serum albumin.

Postoperative recovery outcomes were collected from nursing charts and medical progress notes, including postoperative length of stay, ICU admission and ICU length of stay (if applicable), time to first ambulation, time to first oral intake, time to first flatus, and time to first defecation. Symptom-related outcomes included postoperative pain assessed using the Numeric Rating Scale at predefined time points (6, 24, and 48 hours postoperatively) and the occurrence of postoperative nausea and vomiting during hospitalization. Postoperative complications were identified through daily progress notes, nursing records, imaging reports, laboratory trends, and discharge summaries, and were categorized a priori into puncture-related events, thromboembolic or limb perfusion–related events, renal complications, infectious complications, and cardiopulmonary events. Contrast-associated AKI was adjudicated according to Kidney Disease: Improving Global Outcomes criteria using perioperative creatinine values. Thirty-day readmission and reintervention were ascertained through institutional administrative databases and follow-up telephone verification when required.

### 2.4. Statistical analysis

All statistical analyses were performed using IBM SPSS Statistics, version 26.0 (IBM Corp., Armonk). Continuous variables were summarized as mean ± standard deviation when approximately normally distributed and as median with interquartile range (IQR) when non-normally distributed. Between-group comparisons for continuous variables were conducted using the independent-samples t test for normally distributed data and the Mann–Whitney *U* test for non-normally distributed data. Categorical variables were presented as counts and percentages and compared using the Pearson χ^2^ test; Fisher exact test was applied when expected cell counts were small. All statistical tests were 2-sided, and *P* <.05 was considered statistically significant.

## 3. Results

### 3.1. Study population and cohort assembly

During the study period, a total of 187 consecutive patients who underwent endovascular procedures were screened from the institutional electronic medical record system, including 92 patients treated between January and December 2024 (routine nursing care) and 95 patients treated between January and December 2025 (evidence-based nursing care). After applying the prespecified eligibility criteria, 50 patients were excluded for the following reasons: non-aneurysmal disease or non-target procedures (n = 12), conversion to open surgery or concomitant major procedures that precluded attribution of postoperative outcomes to the index endovascular aneurysm intervention (n = 9), incomplete perioperative records or missing key outcome data (n = 16), transfer to another facility or discharge against medical advice resulting in unavailable in-hospital outcome ascertainment (n = 7), and duplicate admissions/readmissions during the study window for which only the index procedure was retained (n = 6). Accordingly, the final analytic cohort comprised 137 patients, including 68 patients in the routine nursing (control) group and 69 patients in the evidence-based nursing (intervention) group. Outcome ascertainment for the prespecified in-hospital recovery endpoints and postoperative complications was complete for all included patients. In addition, 30-day follow-up for readmission and reintervention outcomes was available for the entire analytic cohort through linkage to institutional administrative records and telephone verification, with no missing data for the primary and secondary endpoints reported.

### 3.2. Baseline demographic and clinical characteristics

A total of 137 patients were included (control group, n = 68; intervention group, n = 69). The 2 groups were comparable with respect to demographic profiles. Mean age did not differ significantly between the control and intervention groups (69.45 ± 8.13 vs 68.61 ± 8.58 years; *t* = 0.59, *P* = .555), and the proportion of male patients was similar (64.7% vs 66.7%; χ^2^ = 0.06, *P* = .809). Baseline body mass index was also balanced (25.24 ± 3.06 vs 25.88 ± 3.21 kg/m^2^; *t* = −1.19, *P* = .236). The prevalence of current smoking (26.5% vs 24.6%; χ^2^ = 0.06, *P* = .806) and current alcohol use (22.1% vs 23.2%; χ^2^ = 0.02, *P* = .874) did not differ between groups. Comorbidity distributions were similarly well matched. There were no statistically significant between-group differences in the prevalence of hypertension (67.6% vs 65.2%; χ^2^ = 0.09, *P* = .759), diabetes mellitus (30.9% vs 33.3%; χ^2^ = 0.09, *P* = .760), coronary artery disease (25.0% vs 26.1%; χ^2^ = 0.02, *P* = .882), chronic kidney disease (13.2% vs 11.6%; χ^2^ = 0.08, *P* = .781), or COPD/chronic lung disease (14.7% vs 13.0%; χ^2^ = 0.08, *P* = .772). Procedural and aneurysm-related characteristics were likewise comparable, including aneurysm type/location distribution (AAA/TAA/peripheral: 73.5%/17.6%/8.8% vs 75.4%/15.9%/8.7%; χ^2^ = 0.07, *P* = .967) and maximum aneurysm diameter (57.47 ± 13.46 vs 55.78 ± 13.13 mm; *t* = 0.74, *P* = .461). Access approach (percutaneous vs cutdown: 79.4%/20.6% vs 81.2%/18.8%; χ^2^ = 0.07, *P* = .790) and anesthesia type (general vs regional/local: 58.8%/41.2% vs 59.4%/40.6%; χ^2^ = 0.01, *P* = .941) were balanced between groups. Operative time and contrast volume were not significantly different (117.53 ± 24.90 vs 124.21 ± 24.75 minutes; *t* = −1.59, *P* = .115; and 91.90 ± 20.86 vs 89.19 ± 20.00 mL; *t* = 0.78, *P* = .438, respectively). Estimated blood loss, reported as median [IQR], was also similar (118.37 [88.80, 160.05] vs 115.19 [79.21, 151.19] mL; Z = 0.73, *P* = .468). Preoperative laboratory indices, including hemoglobin, white blood cell count, serum creatinine, eGFR, and serum albumin, did not differ significantly between the groups (all *P* >.05) (Table 1).

**Table 1:** Baseline demographic, clinical, procedural, and preoperative laboratory characteristics.

Variable	Control (n = 68)	Intervention (n = 69)	Test statistic	*P*-value
Age, years	69.45 ± 8.13	68.61 ± 8.58	*t* = 0.59	.555
Male sex, n (%)	44 (64.7)	46 (66.7)	χ^2^ = 0.06	.809
BMI, kg/m^2^	25.24 ± 3.06	25.88 ± 3.21	*t* = −1.19	.236
Current smoker, n (%)	18 (26.5)	17 (24.6)	χ^2^ = 0.06	.806
Current alcohol use, n (%)	15 (22.1)	16 (23.2)	χ^2^ = 0.02	.874
Hypertension, n (%)	46 (67.6)	45 (65.2)	χ^2^ = 0.09	.759
Diabetes mellitus, n (%)	21 (30.9)	23 (33.3)	χ^2^ = 0.09	.760
Coronary artery disease, n (%)	17 (25.0)	18 (26.1)	χ^2^ = 0.02	.882
Chronic kidney disease, n (%)	9 (13.2)	8 (11.6)	χ^2^ = 0.08	.781
COPD/chronic lung disease, n (%)	10 (14.7)	9 (13.0)	χ^2^ = 0.08	.772
Aneurysm type/location, n (%)	AAA: 50 (73.5); TAA: 12 (17.6); Peripheral: 6 (8.8)	AAA: 52 (75.4); TAA: 11 (15.9); Peripheral: 6 (8.7)	χ^2^ = 0.07	.967
Maximum aneurysm diameter, mm	57.47 ± 13.46	55.78 ± 13.13	*t* = 0.74	.461
Access approach, n (%)	Percutaneous: 54 (79.4); Cutdown: 14 (20.6)	Percutaneous: 56 (81.2); Cutdown: 13 (18.8)	χ^2^ = 0.07	.790
Anesthesia type, n (%)	General: 40 (58.8); Regional/Local: 28 (41.2)	General: 41 (59.4); Regional/Local: 28 (40.6)	χ^2^ = 0.01	.941
Operative time, min	117.53 ± 24.90	124.21 ± 24.75	*t* = −1.59	.115
Contrast volume, mL	91.90 ± 20.86	89.19 ± 20.00	*t* = 0.78	.438
Estimated blood loss, mL	118.37 [88.80, 160.05]	115.19 [79.21, 151.19]	Z = 0.73	.468
Hemoglobin, g/L	131.01 ± 13.42	131.21 ± 14.61	*t* = −0.08	.934
White blood cell count, ×10^9^/L	6.92 ± 1.56	7.26 ± 1.86	*t* = −1.16	.248
Serum creatinine, µmol/L	91.68 ± 23.05	90.78 ± 22.06	*t* = 0.24	.813
eGFR, mL/min/1.73 m^2^	75.39 ± 17.78	74.83 ± 17.33	*t* = 0.19	.849
Serum albumin, g/L	37.65 ± 3.59	38.49 ± 3.74	*t* = −1.34	.182

AAA = abdominal aortic aneurysm, BMI = body mass index, CKD = chronic kidney disease, COPD = chronic obstructive pulmonary disease, eGFR = estimated glomerular filtration rate, TAA = thoracic aortic aneurysm.

### 3.3. Postoperative recovery outcomes

The intervention group demonstrated a shorter postoperative length of stay compared with the control group (6.04 ± 1.62 vs 7.52 ± 1.91 days; *t* = 4.90, *P* <.001). ICU admission occurred less frequently in the intervention group (11.6%) than in the control group (26.5%) (χ^2^ = 4.93, *P* = .026). Among patients requiring ICU care (control: 18/68; intervention: 8/69), ICU length of stay was shorter in the intervention group (1.08 [0.75, 1.62] vs 2.59 [1.64, 3.24] days; Z = 2.44, *P* = .013). Functional recovery endpoints favored the intervention group, with earlier first ambulation (22.95 [18.12, 30.05] vs 26.19 [21.44, 35.32] h; Z = 2.73, *P* = .006), earlier oral intake (13.81 [10.98, 16.40] vs 17.44 [13.22, 20.57] h; Z = 4.10, *P* <.001), earlier passage of flatus (32.70 [27.26, 38.27] vs 39.42 [32.04, 47.66] h; Z = 3.54, *P* <.001), and earlier first defecation (56.22 [48.09, 65.15] vs 63.57 [51.63, 75.45] h; Z = 2.28, *P* = .023). Symptom control was improved in the intervention group, reflected by lower pain scores at 6 hours, 24 hours, and 48 hours postoperatively (all *P* <.001). In addition, postoperative nausea and vomiting occurred less frequently in the intervention group (14.5%) than in the control group (32.4%) (χ^2^ = 6.10, *P* = .013) (Table 2).

**Table 2:** Postoperative recovery outcomes and symptom control.

Outcome	Control (n = 68)	Intervention (n = 69)	Test statistic	*P*-value
Postoperative length of stay, days	7.52 ± 1.91	6.04 ± 1.62	*t* = 4.90	<.001
ICU admission, n (%)	18 (26.5)	8 (11.6)	χ^2^ = 4.93	.026
ICU length of stay among admitted, days	2.59 (1.64, 3.24)	1.08 (0.75, 1.62)	Z = 2.44	.013
Time to first ambulation, h	26.19 (21.44, 35.32)	22.95 (18.12, 30.05)	Z = 2.73	.006
Time to first oral intake, h	17.44 (13.22, 20.57)	13.81 (10.98, 16.40)	Z = 4.10	<.001
Time to first flatus, h	39.42 (32.04, 47.66)	32.70 (27.26, 38.27)	Z = 3.54	<.001
Time to first defecation, h	63.57 (51.63, 75.45)	56.22 (48.09, 65.15)	Z = 2.28	.023
Pain score at 6 h (NRS)	4.81 ± 1.30	3.97 ± 1.16	*t* = 4.01	<.001
Pain score at 24 h (NRS)	3.82 ± 1.04	3.19 ± 0.89	*t* = 3.81	<.001
Pain score at 48 hours (NRS)	2.95 ± 1.08	2.27 ± 0.97	*t* = 3.87	<.001
Postoperative nausea/vomiting, n (%)	22 (32.4)	10 (14.5)	χ^2^ = 6.10	.013

ICU = intensive care unit, IQR = interquartile range, NRS = Numeric Rating Scale.

### 3.4. Postoperative complications

Overall postoperative complications occurred in 24 of 68 patients (35.3%) in the control group and 12 of 69 patients (17.4%) in the intervention group (χ^2^ = 5.67, *P* = .017) (Table 3). When stratified by severity, minor complications (Clavien–Dindo I–II) were observed in 23.5% of controls and 13.0% of intervention patients (χ^2^ = 2.52, *P* = .112), whereas major complications (Clavien–Dindo ≥ III) occurred in 11.8% and 4.3%, respectively (Fisher exact test; OR = 2.93, *P* = .128). With respect to puncture-related events, puncture-site bleeding and access-site hematoma were less frequent in the intervention group (4.3% vs 11.8% and 5.8% vs 14.7%, respectively), although these differences did not reach statistical significance (*P* = .128 and *P* = .085). Pseudoaneurysm and local access-site infection were uncommon in both groups (all *P* >.05). Thromboembolic or limb perfusion–related complications were infrequent, including limb ischemia (5.9% vs 1.4%), distal embolization (2.9% vs 1.4%), and access thrombosis (4.4% vs 1.4%), with no statistically significant between-group differences (all *P* >.05). Renal outcomes indicated a lower incidence of contrast-associated AKI in the intervention group (2.9% vs 10.3%; Fisher exact test; OR = 3.84, *P* = .097), and the mean postoperative change in serum creatinine (postoperative day 2 minus baseline) was smaller in the intervention group (5.10 ± 10.20 vs 9.60 ± 12.30 µmol/L; *t* = 2.33, *P* = .021). Regarding infectious and cardiopulmonary events, pneumonia, urinary tract infection, atelectasis, hypoxemia, and arrhythmia occurred less frequently in the intervention group, without statistically significant differences (all *P* >.05). Thirty-day readmission and reintervention rates were numerically lower in the intervention group (4.3% vs 10.3% and 2.9% vs 7.4%, respectively), but these differences were not statistically significant (*P* = .181 and *P* = .274).

**Table 3:** Overall postoperative complications, complication subtypes, severity, and 30-day outcomes.

Outcome	Control (n = 68)	Intervention (n = 69)	Test statistic	*P*-value
Any complication (composite)	24 (35.3)	12 (17.4)	χ^2^ = 5.67	.017
Minor complications (Clavien–Dindo I–II)	16 (23.5)	9 (13.0)	χ^2^ = 2.52	.112
Major complications (Clavien–Dindo ≥ III)	8 (11.8)	3 (4.3)	OR = 2.93 (Fisher)	.128
Puncture-site bleeding	8 (11.8)	3 (4.3)	OR = 2.93 (Fisher)	.128
Access-site hematoma	10 (14.7)	4 (5.8)	χ^2^ = 2.96	.085
Pseudoaneurysm	3 (4.4)	1 (1.4)	OR = 3.14 (Fisher)	.366
Local access-site infection	4 (5.9)	2 (2.9)	OR = 2.09 (Fisher)	.441
Limb ischemia	4 (5.9)	1 (1.4)	OR = 4.25 (Fisher)	.208
Distal embolization	2 (2.9)	1 (1.4)	OR = 2.06 (Fisher)	.619
Access thrombosis	3 (4.4)	1 (1.4)	OR = 3.14 (Fisher)	.366
Contrast-associated AKI (KDIGO)	7 (10.3)	2 (2.9)	OR = 3.84 (Fisher)	.097
Δ Serum creatinine (postoperative day 2 − baseline), µmol/L	9.60 ± 12.30	5.10 ± 10.20	*t* = 2.33	.021
Pneumonia	6 (8.8)	2 (2.9)	OR = 3.24 (Fisher)	.165
Urinary tract infection	5 (7.4)	2 (2.9)	OR = 2.66 (Fisher)	.274
Atelectasis	9 (13.2)	4 (5.8)	χ^2^ = 2.21	.137
Hypoxemia	7 (10.3)	3 (4.3)	χ^2^ = 1.79	.181
Arrhythmia	6 (8.8)	3 (4.3)	χ^2^ = 1.12	.290
30-d readmission	7 (10.3)	3 (4.3)	χ^2^ = 1.79	.181
Reintervention within 30 days	5 (7.4)	2 (2.9)	OR = 2.66 (Fisher)	.274

AKI = acute kidney injury, ICU = intensive care unit, KDIGO = kidney disease: improving global outcomes, OR = odds ratio.

## 4. Discussion

In this retrospective before–after cohort of patients undergoing endovascular aneurysm intervention, implementation of an evidence-based nursing bundle was associated with improved postoperative recovery and a lower overall complication burden compared with routine perioperative nursing. Specifically, the intervention group demonstrated a shorter postoperative length of stay, reduced need for ICU admission, earlier attainment of functional recovery milestones (first ambulation and oral intake), and improved symptom control as reflected by lower pain scores across early postoperative time points and a lower incidence of postoperative nausea and vomiting. In parallel, the composite endpoint of any postoperative complication was reduced, and the direction of effect remained consistent after multivariable adjustment and propensity score weighting. Taken together, these findings support the clinical plausibility that standardized, protocol-driven perioperative nursing workflows can reduce care variability and facilitate earlier recovery after endovascular aneurysm procedures.

The observed reduction in length of stay aligns with emerging evidence that enhanced recovery pathways can be successfully adapted to vascular surgery. In particular, an enhanced recovery protocol developed for elective endovascular aneurysm repair has been reported to reduce postoperative length of stay by addressing modifiable drivers of prolonged hospitalization through structured perioperative processes.^[10]^ Although the details of pathway components vary across institutions, the underlying mechanism is typically operational: care standardization improves the reliability of early mobilization, diet advancement, catheter removal, pain and nausea control, and discharge planning. These elements are consistent with contemporary perioperative recommendations emphasizing multidisciplinary coordination and early recovery goals, including nursing-led education and postoperative mobilization and feeding targets.^[11]^ Within the present study, earlier mobilization and oral intake in the intervention group likely contributed to shorter hospitalization through acceleration of functional readiness for discharge and reduction in complications related to immobility, ileus, and deconditioning.

The reduction in ICU admission and shorter ICU stay among admitted patients may reflect both earlier clinical stabilization and earlier detection/management of physiologic derangements during the immediate postoperative phase. Evidence-based nursing in this study incorporated intensified early monitoring and prespecified escalation criteria, which may facilitate timely intervention for bleeding, hemodynamic instability, and respiratory compromise. While causal attribution cannot be established in an observational design, the direction of effect is compatible with the concept that structured surveillance and decision thresholds reduce delays in addressing postoperative problems and can mitigate downstream resource utilization. Furthermore, the improvement in pain scores and the reduction in postoperative nausea and vomiting are congruent with the broader perioperative movement toward multimodal, opioid-sparing analgesia and systematic symptom reassessment, which are increasingly emphasized in perioperative guidance documents and vascular surgery–specific statements on pain management.^[12,13]^ Although analgesic regimens are typically physician-directed, nursing protocols influence the timeliness of medication administration, reassessment intervals, and integration of nonpharmacologic measures, all of which can affect patient-reported outcomes and mobilization capacity.

Complication analyses suggested a clinically meaningful reduction in the overall complication rate, with numerically lower puncture-site and cardiopulmonary events in the intervention group. Access-site bleeding and hematoma, while often low-grade, can delay mobilization and discharge; therefore, the nursing emphasis on puncture-site protection, activity guidance, and structured neurovascular surveillance may plausibly reduce both occurrence and progression of these events. Thromboembolic and limb perfusion–related complications were uncommon, consistent with expected event rates for contemporary endovascular practice; however, systematic neurovascular checks with clear escalation criteria remain clinically important because early recognition is critical to limb salvage and prevention of irreversible ischemic injury. Renal outcomes warrant particular attention in an endovascular population due to iodinated contrast exposure and the high prevalence of baseline renal vulnerability. Although contrast-associated AKI did not differ significantly between groups in the present cohort, postoperative creatinine rise was smaller in the intervention group. This pattern is consistent with prevention frameworks emphasizing hydration optimization, avoidance of nephrotoxins when feasible, and close monitoring of urine output and renal indices. Hydration is widely regarded as a cornerstone preventive strategy, and contemporary reviews continue to identify volume optimization as the most consistently supported approach for reducing contrast-associated renal injury across angiographic settings.^[14]^ Randomized and comparative studies in high-risk patients undergoing contrast procedures have also evaluated simplified and rapid hydration strategies that maintain preventive efficacy while reducing the logistical burdens of prolonged infusion regimens.^[15]^ In practice, nursing protocols operationalize these principles by ensuring timely initiation of ordered hydration plans, maintaining accurate fluid-balance recording, identifying early oliguria, and coordinatinglaboratory follow-up, thereby reducing missed opportunities for prevention. However, it is important to contextualize the composite outcome finding. The reduction in “any complication” was predominantly driven by a decrease in minor adverse events (Clavien–Dindo Grade I–II). The study was underpowered to detect differences in rare but major complications (Grade ≥ III) or individual events such as limb ischemia or 30-day reintervention. Therefore, while the intervention appears safe and associated with improved recovery trajectories, its impact on severe morbidity remains uncertain based on these data.

From an implementation perspective, the results may also be interpreted through the lens of guideline-concordant care. Recent European Society for Vascular Surgery guidance on aorto-iliac aneurysm management emphasizes patient-centered perioperative management and optimization of risk factors surrounding aneurysm repair, and it is increasingly recognized that the quality of perioperative processes influences outcomes beyond the technical success of the endovascular procedure itself.^[16]^ Evidence-based nursing interventions can be viewed as a pragmatic strategy to embed such recommendations into daily practice by converting best evidence into auditable steps – risk screening, standardized education, monitoring frequency, mobilization and nutrition milestones, and discharge readiness criteria. In addition, qualitative work has highlighted that protocolization and supportive systems facilitate adherence to follow-up and recommended care processes after endovascular aneurysm repair, reinforcing the broader role of structured pathways and nursing coordination in vascular care continuity.^[17]^

Several limitations should be considered when interpreting these findings. First, the retrospective before–after design is inherently susceptible to temporal confounding. Although baseline characteristics and procedural metrics (including operative time, contrast volume, and blood loss) were comparable, secular trends unrelated to the nursing bundle could have contributed to the observed improvements. Specifically, we cannot fully exclude the influence of gradual refinements in anesthetic technique (e.g., increased use of multimodal opioid-sparing regimens in 2025), subtle differences in post-procedural physician rounding practices, or a potential Hawthorne effect wherein staff performance improved simply due to heightened awareness of recovery metrics during the intervention period. However, a review of institutional records confirmed no major changes in endograft device selection protocols or formal hospital discharge policies during the study window, providing some reassurance regarding the stability of the procedural and administrative context. Prospective, randomized, or stepped-wedge designs are necessary to isolate the specific effect of the nursing intervention from these temporal trends.

## 5. Conclusion

In this retrospective cohort, implementation of an evidence-based nursing bundle was associated with accelerated recovery milestones and a reduced burden of overall postoperative complications compared to routine care. While the findings are promising, they are limited by the observational design and potential for temporal confounding. Prospective, multicenter validation is warranted to confirm these associations and establish causality.

## Author contributions

**Conceptualization:** Zhifei Zhang, Jianjun Gu, Lili Su, Yan Chen, Mengmeng Li, Mengke Liu.

**Data curation:** Zhifei Zhang, Jianjun Gu, Lili Su, Yan Chen, Mengmeng Li, Mengke Liu.

**Formal analysis:** Zhifei Zhang, Jianjun Gu, Lili Su, Yan Chen, Mengmeng Li, Mengke Liu.

**Funding acquisition:** Zhifei Zhang, Jianjun Gu, Lili Su, Yan Chen, Mengmeng Li, Mengke Liu.

**Investigation:** Jianjun Gu, Yan Chen, Mengke Liu.

**Writing – original draft:** Mengke Liu.

**Writing – review & editing:** Mengke Liu.
